# The Emergence of National Electronic Health Record Architectures in the United States and Australia: Models, Costs, and Questions

**DOI:** 10.2196/jmir.7.1.e3

**Published:** 2005-03-14

**Authors:** Tracy D Gunter, Nicolas P Terry

**Affiliations:** ^2^Center for Health Law StudiesSchool of LawSaint Louis UniversitySt. Louis, MOUSA; ^1^Department of PsychiatryRoy J. and Lucille A. Carver College of MedicineUniversity of IowaIowa City, IAUSA

**Keywords:** Medical records systems, computerized, delivery of health care, patient care, information management, medical record linkage, confidentiality, policy making, United States, Australia, Internet

## Abstract

Emerging electronic health record models present numerous challenges to health care systems, physicians, and regulators. This article provides explanation of some of the reasons driving the development of the electronic health record, describes two national electronic health record models (currently developing in the United States and Australia) and one distributed, personal model. The US and Australian models are contrasted in their different architectures (“pull” versus “push”) and their different approaches to patient autonomy, privacy, and confidentiality. The article also discusses some of the professional, practical, and legal challenges that health care providers potentially face both during and after electronic health record implementation.

## Introduction

The electronic health record (EHR) is an evolving concept defined as a longitudinal collection of electronic health information about individual patients and populations. Primarily, it will be a mechanism for integrating health care information currently collected in both paper and electronic medical records (EMR) for the purpose of improving quality of care. Although the paradigmatic EHR is a wide-area, cross-institutional, even national construct, the electronic records landscape also includes some distributed, personal, non-institutional models.

Emerging EHR models present numerous challenges to health care systems, physicians, and regulators. This article provides explanation of some of the reasons driving the development of the EHR, describes three different EHR models, and discusses some of the practical and legal challenges that health care providers potentially face both during and after EHR implementation.

### Stakeholders and Drivers

Information technology (IT) has become the principal vehicle that some believe will reduce medical error. In the United States, the non-governmental and highly influential Institute of Medicine (IOM) has committed to technology-led system reform [1] and urged “a renewed national commitment to building an information infrastructure to support health care delivery, consumer health, quality measurement and improvement, public accountability, clinical and health services research, and clinical education.” [2] As is well known, this IT-led system reform involves several intersecting technologies, including the following: tracking systems (barcodes and Radio Frequency Identification [RFID]); computerized physician order entry (CPOE) systems; clinical decision support systems (CDSSs) that complement order entry devices operating with server-side systems that reference drug interaction information or treatment models (such as clinical practice guidelines); and enhanced reporting systems that provide for adverse event and medical error disclosure, and facilitate population-based health care models and more extensive outcomes research.

The electronic record is at the center of the IOM's goal of eliminating most handwritten clinical data by the end of this decade [2]. Electronic records are superior to paper records because they decrease error due to handwriting problems and ease physical storage requirements [3]. Additionally, electronic records simultaneously leverage other error-reducing technologies and render them coherent. EHR models present significant additional advantages because of their potential to deliver a longitudinal record that tracks all medical interactions by a particular patient and provide comprehensive data across populations. Thus, the IOM envisions a longitudinal collection of electronic health information for and about individuals and populations as feeding data into error-reducing “knowledge and decision support systems.” [4,5]

Error reduction aside, business concerns and structural changes in health care delivery are driving EHR implementation. Although some of these phenomena are unique to the US model of health care financing and delivery, mature systems in other countries must also accommodate stresses from similar developments. First, the shift from in-patient to ambulatory care (and other episodic models) has accelerated the need for accurate and efficient flow of patient medical and billing information between organizationally and geographically distinct providers. Second, the operational aspects of managed care, such as the data needs of “gate keeping” physicians, demands by payers for performance “report cards,” and system administrators' increasing needs for sophisticated utilization review and risk management tools, have increased the need for data transparency [6]. Third, the growth of “shared care”, whereby the patient both shares responsibility with the provider for care and is likely to have increasingly fragmented or episodic relationships with multiple providers, requires that patients must have access to health data generally and, more controversially, to information in their record [7,8]. Furthermore, it requires that providers have transparent access to other occasions of treatment, particularly pharmacotherapy. Finally, both patients and regulators are demanding increasing amounts of data regarding errors or near misses and outcomes in populations [9]—data that is difficult to generate without sophisticated data coding and nearly impossible to analyze without complex, comprehensive database systems.

In addition to safe, high-quality care, patients expect privacy, rights of access and correction [7], and the opportunity to give consent for research uses of their health information [10]. As patient care moves from an in-patient to ambulatory or other fragmented models of service delivery utilizing multiple providers, the portability of and timely access to data become increasingly important to patients as well as providers. In the words of one patient,

I don't want much - just for my medical records to be seen only by those whom I authorize, and for the record to be readily accessible to them wherever they are. . . . I would like a bigger say in what goes into my notes, and if I don't like something I would like it taken out. [11]

Providers continue to embrace confidentiality to foster an environment in which patients will disclose information related to their health. However, in the realm of health information, the needs of those delivering, regulating, and paying for health care may be at odds with the principles of privacy and confidentiality [12,13]. Technological acquisition, storage, access to, and distribution of patient health data exacerbates that tension.

In addition to maintaining confidentiality, providers are subject to legal and ethical obligations to evaluate and document the encounter. Providers engage in narrative with the patient and form opinions throughout and across interviews [14]. Therefore it follows that the available EHR vocabulary must accommodate symptoms and modifiers in addition to diagnoses and summary statements [14]. Data entry systems must be seamless and unobtrusive, and should include handwriting or voice recognition in addition to standardized checklists and templates. Otherwise, provider time will be lost as physicians attempt to code findings during the encounter [14]. Since medical care itself is not standardized, it remains difficult to envision a “one size fits all” approach to medical record computing [8,15].

Although there has been debate among providers about the feasibility and safety of having all patient information computerized and available across institutions, the authors accept the premise that EHR implementation is inevitable because of the support for the idea from health care regulators, third-party payers, hospital administrators, and physician advocacy groups such as the American Medical Association [16].

## Progress and Models

As EHR models have struggled towards maturity, some key questions have arisen. Debatable issues include the following: whether the originating record should supply complete data or a summary; whether the data subsequently generated is episodic or longitudinal; and whether patients and providers will either control which information is “pushed” to the central record or be spectators as comprehensive data is “pulled” by remote systems. The EHR models that are developing in Australia and the United States suggest some divergent answers to these questions. Although less visible than institutional (provider or governmental) models, a third EHR model focuses on a web-based, distributed “personal” longitudinal record. This model raises discrete quality and confidentiality issues.

### Australia

Australia's proposed national health information network is called Health*Connect* [17]. The basic Health*Connect* model is to extract a summary record from locally collected patient data which is then aggregated to create a centralized Health*Connect* record that may then be shared among participating and authorized providers [18].

A Health*Connect* “event summary” consists of the “critical information considered to be useful to other health care providers involved in the future care of the consumer.” [19] Thus, Health*Connect* does not create a comprehensive longitudinal record. Rather, patients, with their providers, will choose which elements may be extracted from an existing health record and transmitted to the Health*Connect* record. Providers, with the consent of their patients, may subsequently add data to the Health*Connect* record. It follows, therefore, that *HealthConnect* is a “push” system, selectively sending data to a centralized record [20].

The patient controls which elements of the centralized record may be used for which purposes or displayed in which “views” [21]. For example, a patient might elect to include details of his psychotropic prescriptions in an event summary and consent to all his prescribing doctors viewing that data, but only consent to other mental health professionals viewing his psychiatrist's discharge order. The system's dedication to voluntary participation is desirable based on demonstrated patient interest in confidentiality. However, the summary data that is centralized may not fully support the system's secondary goals of disseminating professional education, supporting research, furthering utilization, increasing access, and improving quality [20]. Health*Connect* has completed 2 years of pilot testing. It is estimated that the system will save AUD $300 million per year by reducing errors and duplication of effort [20].

### United States

The IOM has been critical of the rate of technology adoption by US hospitals [22]. Notwithstanding, and representing the public sector, the Department of Veterans Affairs is committed to process reform and technologically mediated delivery of services [23]. More broadly, the Consolidated Health Informatics (CHI) initiative is accelerating the use of common clinical vocabularies and messaging standards across federal agencies that process health data [24]. In addition to projects of national scope, some state governments have EHR launch initiatives; for example, Massachusetts has recently announced a statewide initiative, partially funded by the health insurer Blue Cross Blue Shield, with the goal of having a statewide electronic records system in place within five years [25]. Similar initiatives are being undertaken by some of the largest private providers; for example, Kaiser Permanente, the largest nonprofit health management organization (HMO) in the United States, with some 8.4 million members in 9 states and 12000 participating physicians, has recently adopted a 3-year, $1.8 billion electronic records program [26].Providing additional direction in developing EHR models have been the Connecting for Health initiative funded by the Markle Foundation [27], and the work of the EHR Collaborative [28], which consists of the major professional stakeholders such as the American Medical Association, and the Healthcare Information and Management Systems Society.

In the United States, as is the case in Australia and the UK [29], the purer EHR model is evolving at the national level. To date, the IOM [30] and the National Committee on Vital and Health Statistics (NCVHS) [31,32] have focused primarily on the *technical* aspects of EHR implementation in the United States. Both have identified two core components in the project: first, building a national health information infrastructure and, second, establishing data interoperability and comparability for patient safety data. In order to achieve data interoperability and comparability, NCVHS and IOM have recommended the adoption of core standardized EHR terminologies (eg, ICD-9 for diseases or symptoms [33], CPT-4 to code medical procedures, and services [34], and RxNorm for drug names and doses [35]). Considerable development is also underway to standardize event taxonomy (eg, adverse event or near-miss reporting using the College of American Pathologists' SNOMED CT taxonomy [36]) and to express knowledge representation such as clinical practice guidelines.

At this stage in the development of the US national model, its architects are concentrating on the interoperability and comparability of *all* patient safety-related data [37], designing a full “pull” architecture such that centralized and local records can import semantically similar data. Currently it is unclear which data consumers will choose to extract from remote systems or what limitations will be imposed, or by whom.

### The Internet Alternative—the Personal EHR

Most EHR initiatives are national in scope and frequently government initiated or funded. EMR initiatives are typically hospital- or system-wide, yet are being designed with an eye to broader push or pull systems that will make wide-area use of such institutional data. A personal EHR model is quite different in concept. It assumes that individual patients will aggregate their diverse records and then make them selectively available to new or emergency providers. There are several subscription, web-based personal EHR systems such as PersonalMD.com [38] and Vital Vault [39] that provide secure web space in which patients can aggregate their medical data. Some of these systems also offer automated updating from select providers. Thus, the emerging model emulates popular personal finance applications (such as Microsoft Money or Intuit's Quicken) that allow for both end-user input and importation of data from institutional records to allow management of accounts. As with many emerging Internet-based health-related services, personal EHRs are immature, tend to exhibit limited functionality, and lack permanence [40,41].

## Challenges

While Australia's Health*Connect* respects patient and provider choices and generates only limited data sets, the US system seems to be moving towards interoperability and comparability of *all* patient data, maximizing patient data flow into local and national systems but, arguably, at the cost of patient autonomy. The Australian system may pay too much attention to patient consent and jeopardize broader outcomes and reporting goals. Both institutional systems require careful scrutiny with regard to their costs, confidentiality, and liability risks. The nascent Personal EHR model generates additional concerns, which are similar to those experienced with other web-based products such as medical advice sites.

### Cost

Considerable uncertainty exists regarding the costs associated with electronically mediated health initiatives and their allocation [42]. During transitional periods, costs rise as both traditional and technologically mediated models work in parallel. Most immediately, the health care industry will have to adjust to costs associated with evolving technologies and short system-lives. There has been recent controversy in the United States over Congressional rejection of President Bush's initiative to expand funding for the Office for National Health Information Technology coordination (ONCHIT) of the Department of Health and Human Services; this will likely jeopardize public-sector EHR demonstration projects that were to have been funded out of that office [43].

Equally, there are practical, economic, political, and professional barriers that impede the acceptance of electronic records systems. Individual physicians or small practice groups have particular concerns about the costs and learning curves associated with electronic records systems [44]. Additionally, there are questions about whether to convert records retrospectively or whether electronic records systems should be prospective. Predictably, the medical community is concerned about costly dependence on proprietary technology companies, which could potentially monopolize the hardware and software required for interoperability. One possible solution would be for the mechanism of implementation of the EHR to be a public service built to public standards and/or under patient control [45].

### Privacy and Confidentiality

An EHR system must satisfy its users regarding privacy, confidentiality, and security [46]. In the United States, the Health Insurance Portability and Accountability Act (HIPAA), passed in 1996 [47], committed the federal government to a process of “Administrative Simplification” to reduce health care costs. That mandate included regulatory authority to promulgate national Standards for Privacy of Individually Identifiable Health Information (PIHI) [48]. The PIHI regulations only regulate the disclosure of health data; they place no limitations on its the collection. Although the regulations limit use and disclosure with a “minimum necessary” rule [49], that limitation is inapplicable in cases of treatment or when disclosure is required by law [50]. Further, PIHI permits disclosure to a very broad range of public health, law enforcement, and judicial authorities [51], and provides for less than robust control of disclosures for secondary uses, such as marketing by providers [52]. Confusingly the PIHI regulations only supplement more rigorous state privacy laws. More recently, the HIPAA legislation has given rise to comprehensive federal security rules that govern health care transactions [53].Their limitations, notwithstanding the regulations made under HIPAA, apply to existing health records kept by most providers and are equally applicable to forthcoming EMR and EHR data. It appears unlikely, however, that US EHR developments will be accompanied by any additional protections, either by providing enhanced collection (privacy) or disclosure (confidentiality) rules or by derogating from a pure “pull” model of data aggregation.

Australian state [54] and federal (Commonwealth) governments aggressively protect patient information [55]. The Commonwealth National Privacy Principles [56] are broadly sensitive to the needs of the health information domain and protect patients with collection-centric (by placing limits on collection and granting consumers anonymity rights) and disclosure-centric rules as well as addressing data quality, data security, and access rights. In 2001, the Australian Federal Privacy Commissioner issued his nonbinding but influential initial Guidelines on Privacy in the Private Health Sector [57] that map the National Privacy Principles to the health context and provide for a robust collection-centric approach. In most cases, consent is required prior to collecting patient health information. This consent should include disclosure of the purposes for which the information is being collected. Further, the “[i]nformation collected should be limited to what is necessary for the health service provider's functions and activities.” [58] The Guidelines state that a provider should “only use or disclose personal information for the primary purpose for which it was collected, or for directly related secondary purposes if these fall within the reasonable expectations of the individual” [59]. As a result, the Guidelines provide a satisfactory framework for emerging EHR models, while the Health*Connect* patient-controlled “push” model is intrinsically protective of patient interests.

The US PIHI rules regulating the disclosure of health data have less certain application outside traditional bricks-and-mortar providers, such as those engaged in Internet prescribing and web-based medical advice [60]. As a result, considerable attention needs to be paid to the confidentiality and security of data stored by Personal EHR businesses. In many cases the patient's protection will be limited to that granted by a privacy policy published by the personal EHR provider.

### Litigation Risks

Privacy and confidentiality aside, providers already face legal costs with regard to their records. For example, a US provider's failure to maintain timely, legible, accurate and complete records will likely breach state licensure standards [61,62], with severe disciplinary implications [63,64], and may also jeopardize Medicare participation [65]. Improper record keeping may also give rise to medical malpractice liability [66]. In this context, at least one US court has expressed doubt as to the adequacy of a summary rather than comprehensive record [67].

EHR systems inevitably will contribute other costs for users because of interactions with the legal system. Emerging EHR systems, particularly those linked to CDSSs, will be vulnerable to actions focusing on design or other operational flaws [68]. Providers who adopt immature systems may face liability risks because of system deficiencies or insufficient training; those who wait for mature systems are likely to face actions for their failure to implement new but plaintiff-labeled “state-of-the-art” records and CDSSs [69]. Adoption of electronic records systems may also create more indirect legal costs. Litigants may attempt to leverage the new systems to promote their recovery in clinical negligence cases. For example, plaintiffs' attorneys may attempt to use data-mining tools to identify related occurrences to bolster evidence or use their clients' rights of access and modification to manipulate the patient record [70].

## Conclusion

On April 26, 2004, President Bush announced the goal of assuring that most Americans have EHRs within the next 10 years [71]. To this end, the President appointed a National Health Information Technology Coordinator to guide the “nationwide implementation of interoperable health information technology.” [72]

If properly funded and nationally implemented, the US EHR model has the following potentials: to interconnect with and enhance other error-reducing and cost-saving technologies such as decision support systems; to streamline health care dataflow using an interoperable and standardized nomenclature; to improve quality by encouraging accurate and legible communication among providers; to automate adverse event and medical error disclosure; and to facilitate reliable and reproducible outcomes research and reporting [73].

As EHR progress continues, several important questions remain unanswered. Which is the preferable EHR model—a shared summary system or a full interpretational longitudinal record? How much say will or should patients and providers have regarding which health information is shared across systems? Would an interactive EHR increase patient interest and involvement in their own care? And, of course, will electronic records conquer the technical problems they pose, avoid the security and privacy costs their critics identify, and deliver lower costs and higher quality; or will they be responsible for still more costs and errors, while promoting the continued industrialization of health care delivery and subordinating patient autonomy and professional ideals to soulless systems?

It has never been more important for providers to be aware of emerging technology, to comprehend the tension between improved care and the preservation of patient privacy and autonomy, and to offer feedback to the American Medical Association and other professional bodies as these entities move to influence the development of the EHR.

## Abbreviations

CDSS: computerized decision support system
CHI: Consolidated Health Informatics
CPOE: computerized physician order entry
EHR: electronic health record
EMR: electronic medical record
HIPAA: Health Insurance Portability and Accountability Act
HMO: health management organization
IOM: Institute of Medicine
IT: information technology
NCVHS: National Committee on Vital and Health Statistics
PIHI: Standards for Privacy of Individually Identifiable Health Information
RFID: Radio Frequency Identification
